# Neonatal Treatment with Astaxanthin-Loaded Stealth Solid Lipid Nanoparticles Activates the Impaired NRF2 Pathway and Reduces Hippocampal Oxidative Stress in a Mouse Model of Trisomy 21

**DOI:** 10.3390/cells15161495

**Published:** 2026-08-19

**Authors:** Laura Angelozzi, Debora Santonocito, Francesca Flotta, Beatrice Uguagliati, Marco Emili, Noemí Rueda Revilla, Carmen Martínez-Cué, Carmelo Puglia, Fiorenza Stagni, Sandra Guidi

**Affiliations:** 1 Department for Life Quality Studies, University of Bologna, 47921 Rimini, Italy; laura.angelozzi3@unibo.it; 2 Department of Drug and Health Sciences, University of Catania, 95125 Catania, Italy; debora.santonocito@unict.it (D.S.); capuglia@unict.it (C.P.); 3 Department of Biomedical and Neuromotor Sciences, University of Bologna, 40126 Bologna, Italy; francesca.flotta2@unibo.it (F.F.); beatrice.uguagliati@unibo.it (B.U.); marco.emili2@unibo.it (M.E.); 4 Department of Physiology and Pharmacology, University of Cantabria, 39005 Santander, Spain; noemi.rueda@unican.es (N.R.R.); carmen.martinez-cue@unican.es (C.M.-C.)

**Keywords:** Down syndrome, astaxanthin, NRF2 pathway, oxidative stress

## Abstract

**Highlights:**

**What are the main findings?**

- The NRF2 antioxidant pathway is already impaired during the neonatal period in the hippocampus of Ts65Dn mice.
- Neonatal treatment with astaxanthin-loaded stealth solid lipid nanoparticles (AST-SSLNs) activates the NRF2 pathway and markedly reduces ROS accumulation, lipid peroxidation, and protein oxidation in the hippocampus.

**What are the implications of the main findings?**

- These findings identify the early postnatal period as a potential therapeutic window to target oxidative stress in trisomy 21.
- AST-SSLNs represent a promising and safe nanomedicine-based strategy to counteract early oxidative damage in trisomy 21.

**Abstract:**

Background: Oxidative stress is an important contributor to brain abnormalities in Down syndrome (DS), but the status of the nuclear factor erythroid 2-related factor 2 (NRF2) antioxidant pathway during early postnatal development remains poorly understood. The current study aimed to investigate whether an impairment of the NRF2 pathway is already present in the Ts65Dn mouse model of trisomy 21 at neonatal life stages and whether early treatment with astaxanthin-loaded stealth solid lipid nanoparticles (AST-SSLNs) positively impacts NRF2 signaling and reduces oxidative stress. Methods: Hippocampal NRF2 pathway components and oxidative stress markers were analyzed in neonate Ts65Dn and euploid mice. From postnatal day (P)3 to P15, mice received daily subcutaneous injections of AST-SSLNs or unloaded nanoparticles. NRF2 pathway activation, reactive oxygen species (ROS), lipid peroxidation, protein carbonylation, and safety parameters were evaluated. Results: Untreated Ts65Dn mice exhibited early impairment of the NRF2 pathway, characterized by increased BACH1, reduced NRF2 activation, and decreased HO-1 expression. Neonatal AST-SSLN treatment enhanced NRF2 activation, improved HO-1 levels, and normalized ROS accumulation, lipid peroxidation, and protein carbonylation in the hippocampus, a brain region critically impaired in DS. Treatment had no adverse effects on survival, body weight, or brain weight. Conclusions: These findings demonstrate that NRF2 pathway dysfunction is an early event in trisomy 21 and identify the neonatal period as a potential therapeutic window to counteract oxidative stress. AST-SSLNs represent a promising nanomedicine-based strategy to activate the impaired NRF2 pathway and reduce early hippocampal oxidative damage in DS.

## 1. Introduction

Trisomy 21, clinically recognized as Down syndrome (DS), is the most common chromosomal disorder associated with intellectual disability and is caused by the complete or partial triplication of human chromosome 21 (HSA21) [1,2]. The presence of an extra copy of HSA21 results in a gene dosage imbalance that profoundly affects brain development and function, leading to a broad spectrum of neurological, cognitive, and behavioral abnormalities [3,4]. Individuals with DS exhibit characteristic impairments in learning and memory, reduced cognitive performance, delayed language acquisition, and deficits in motor skills [5,6]. Neuropathological studies have reported that the DS brain is characterized by widespread hypotrophy, reduced neuronal density, impaired neurogenesis, reduced dendritic complexity, abnormal synaptic connectivity, and altered neurotransmission [3,7,8,9]. Importantly, many of these abnormalities emerge during fetal life and progressively worsen with aging, ultimately contributing to the early onset of Alzheimer-like neurodegeneration observed in most individuals with DS [8].

Among the mechanisms implicated in DS pathogenesis, oxidative stress (OS) plays an important role in both neurodevelopmental impairment and early-onset neurodegeneration. OS is defined as an imbalance between oxidants and antioxidants in favor of the former, leading to disruption of redox signaling and/or molecular damage [10,11]. The brain is highly vulnerable to oxidative damage because of its high oxygen consumption, elevated lipid content, and relatively low antioxidant capacity [12,13]. In trisomy 21, OS is multifactorial. Indeed, it results from (i) the overexpression of several HSA21 genes that are directly or indirectly involved in redox regulation, including superoxide dismutase 1 (SOD1), amyloid precursor protein (APP), BTB and CNC Homology 1 (BACH1), and S100 beta (S100 β); (ii) low levels of antioxidants and reducing agents; and (iii) mitochondrial structural and functional abnormalities [14]. These alterations contribute to increased OS levels in trisomy 21, leading to modifications of lipids, proteins, and DNA, which result in lipid peroxidation, protein oxidation, and DNA damage [15]. Most studies have investigated OS in DS individuals and preclinical models at adult life stages [15]. Some evidence indicates that in trisomy 21, OS begins during prenatal life stages. A redox proteomics analysis of the amniotic fluid of women carrying fetuses with DS showed increased oxidative stress. Importantly, the study by Odetti et al. found elevated levels of lipid and protein oxidation in the brains of fetuses with DS in comparison with a control group [16]. These results suggest that redox imbalance represents a key pathogenic mechanism underlying alterations in brain development, as it interferes with crucial processes such as neurogenesis, neuronal maturation, and synaptic function [17].

A master regulator of the endogenous antioxidant response is the Nuclear factor erythroid 2-related factor 2 (NRF2) pathway [18]. NRF2 is a transcription factor that controls the expression of numerous antioxidants and cytoprotective genes, such as heme oxygenase-1 (HO-1), nicotinamide adenine dinucleotide phosphate (NAD(P)H), quinone oxidoreductase 1 (NQO1), and several glutathione-related enzymes. Under physiological conditions, NRF2 is retained in the cytoplasm by Kelch-like ECH-associated protein 1 (KEAP1) and undergoes rapid proteasomal degradation. In response to OS, NRF2 dissociates from KEAP1, translocates into the nucleus, and drives the transcription of antioxidant genes via antioxidant response elements (AREs) [18]. Recent evidence demonstrated that this protective pathway is impaired in the brains of 2-month-old Ts2Cje mice, a model of trisomy 21, as well as in human DS lymphoblastoid cell lines and in brain samples of adults with DS [19,20]. This impairment was found to be associated with the overexpression of BACH1. This gene, located on HSA21, codes for a transcriptional repressor that interferes with NRF2 activity by competing for ARE binding sites, thereby suppressing antioxidant gene transcription. The impairment of this antioxidant response has been shown to result in the accumulation of oxidative damage [21]. Interestingly, BACH1 has been found to be overexpressed in the cortex of DS fetuses [22], suggesting that it may interfere with the activation of the NRF2 pathway at early life stages.

Among compounds with remarkable antioxidant and anti-inflammatory activity, astaxanthin (AST) has recently gained increasing attention. AST is a naturally occurring xanthophyll carotenoid found in microalgae, salmon, shrimp, and other marine organisms [23,24]. Several studies have demonstrated the efficacy of AST in treating and preventing various pathologies, including those of the central nervous system [25]. Specifically, AST has shown beneficial and neuroprotective effects in cerebral ischemia [26] and in chronic neurodegenerative diseases such as Alzheimer’s and Parkinson’s diseases [27,28,29]. Interestingly, the high antioxidant power of AST, which is 6000 times greater than that of vitamin C and 100 times greater than that of vitamin E [30], is attributed not only to its ability to trap reactive oxygen species (ROS) within its polyene chain or neutralize them through chemical bonds, but also to its capacity to modulate the NRF2 signaling pathway. Indeed, AST has been shown to promote the translocation of NRF2 into the nucleus, with a consequent increase in the expression of enzymes involved in the antioxidant response in different models of brain aging and damage [31,32]. Despite its promising biological properties, the therapeutic application of AST is limited by its poor water solubility, chemical instability, and low bioavailability, which limit its systemic distribution. In addition, AST is highly susceptible to oxidation and degradation upon exposure to light, oxygen, or heat—factors that further reduce its efficacy in vivo. To overcome these limitations, innovative nanotechnology-based delivery systems have been developed to improve the stability and bioavailability of AST. In particular, lipid-based nanocarriers, such as stealth solid lipid nanoparticles (SSLNs), represent a promising tool for enhancing the pharmacokinetic profile and therapeutic efficacy of AST. The encapsulation of AST into SSLNs protects the molecule from degradation, prolongs circulation time, improves tissue distribution, and facilitates its passage across the blood-brain barrier, thereby increasing its potential neuroprotective effects [33].

In vivo preclinical data on the NRF2 pathway at early life stages are still lacking in trisomy 21. Furthermore, the potential of AST to counteract oxidative stress has never been explored in this genetic condition. To fill this gap, in the current study we sought to establish whether the NRF2 pathway is already compromised during neonatal life and explored the possibility that neonatal treatment with SSLNs loaded with AST is able to modulate this pathway and reduce oxidative stress. To this purpose, we have exploited the Ts65Dn model of DS. This is the most widely used and extensively characterized mouse model of trisomy 21 as it recapitulates several neuropathological and behavioral features observed in individuals with DS, such as increased OS, impaired neurogenesis, dendritic and synaptic abnormalities, altered neurotransmitter systems, and severe deficits in learning and memory tasks [34,35,36,37]. We focused on the hippocampus, a brain region that has been reported to be highly susceptible to OS and is critically involved in learning and memory processes, particularly in the acquisition of new information, spatial navigation, and consolidation of long-term memories.

## 2. Materials and Methods

### 2.1. Colony

Ts65Dn mice were generated by mating B6EiC3Sn a/A-Ts(17^16^)65Dn females (1924 line) with C57BL/6JEiJ × C3H/HeSnJ (B6EiC3Sn) F1 hybrid males. The parental generation was provided by Jackson Laboratories (Bar Harbor, ME, USA). To maintain the original genetic background and minimize the risk of genetic drift, we used first-generation offspring from this breeding for treatment and introduced new Ts65Dn females into the parental colony approximately every ~6 months from the Jackson Laboratory. Animals were genotyped as previously described [38]. The day of birth was designated as postnatal day zero (P0). The animals’ health and comfort were controlled by the veterinary service. The animals had access to water and food ad libitum and lived in a room with a 12:12 h light/dark cycle. Experiments were performed in accordance with the European Communities Council Directive of 24 November 1986 (86/609/EEC) for the use of experimental animals and were approved by the Italian Ministry of Public Health (212/2024-PR). All efforts were made to minimize animal suffering and to keep the number of animals used to a minimum.

### 2.2. SSLNs Preparation

AST-SSLNs were prepared by the solvent-diffusion technique according to a method previously developed by our group [33], with slight modifications. Briefly, stearic acid (0.005 g; Merck, Milan, Italy) and astaxanthin (4.2 mg; Carbosynth, Compton, Berkshire, UK) were dissolved in ethanol (1.2 mL; Merck) and maintained at 70 °C. The aqueous phase consisted of hydroxypropyl methylcellulose (0.05 g, HPMC K15M; Merck), soy lecithin (0.05 g; Nikko Chemical, Milan, Italy), Lutrol F68^®^ (0.05 g; BASF Chem-Trade GmbH, Burgbernheim, Germany), and distilled water (5.8 mL). The lipid phase was dispersed into the aqueous phase under magnetic stirring at 1400 rpm for 8 min, followed by ultrasonication for 10 min (UP 400 S, Dr. Hielscher GmbH, Stuttgart, Germany). The resulting dispersion was cooled in an ice bath for 5 min. Subsequently, polysorbate 80 (20% *w*/*w* relative to the lipid phase; Polichimica S.r.l., Bologna, Italy) was added under magnetic stirring (250 rpm for 30 min) for surface modification. Finally, the organic solvent was removed under vacuum.

The physicochemical characterization of AST-SSLNs was performed according to methods previously reported by our group [39]. The average particle size (Z-Ave), polydispersity index (PDI), and zeta potential (ZP) were determined using a Zetasizer Nano-ZS90 (Malvern Instruments Ltd., Malvern, UK). Samples were diluted (100 μL in 900 μL of distilled water) before analysis. Measurements were carried out at 20 ± 0.2 °C, and all analyses were performed in triplicate.

### 2.3. Experimental Protocol

To characterize the NRF2 pathway in the neonatal period, we used hippocampi previously dissected from the right hemisphere of P15 euploid and Ts65Dn mice that were stored at −80 °C in our laboratory (total number of analyzed mice: euploid *n* = 13, 5 females, 8 males; Ts65Dn *n* = 13, 4 females, 9 males). The animals of this group are referred to as “untreated”.

All euploid and Ts65Dn mice that survived during the period from P0 to P3 entered the study of the effects of AST, with no specific selection criteria, and following genotyping, they were randomly assigned to the experimental groups. Investigators administering the treatments were aware of the genotype and treatment allocation. However, tissue processing, sample preparation, outcome assessment, and data analysis were performed by investigators blinded to the experimental group allocation. An a priori sample size calculation was performed using G*Power software 3.1.9.7 to determine the minimum number of animals required per experimental group, assuming a significance level of α = 0.05 and a statistical power of 80%. The expected effect size was estimated from previous studies.

**Figure 1:**
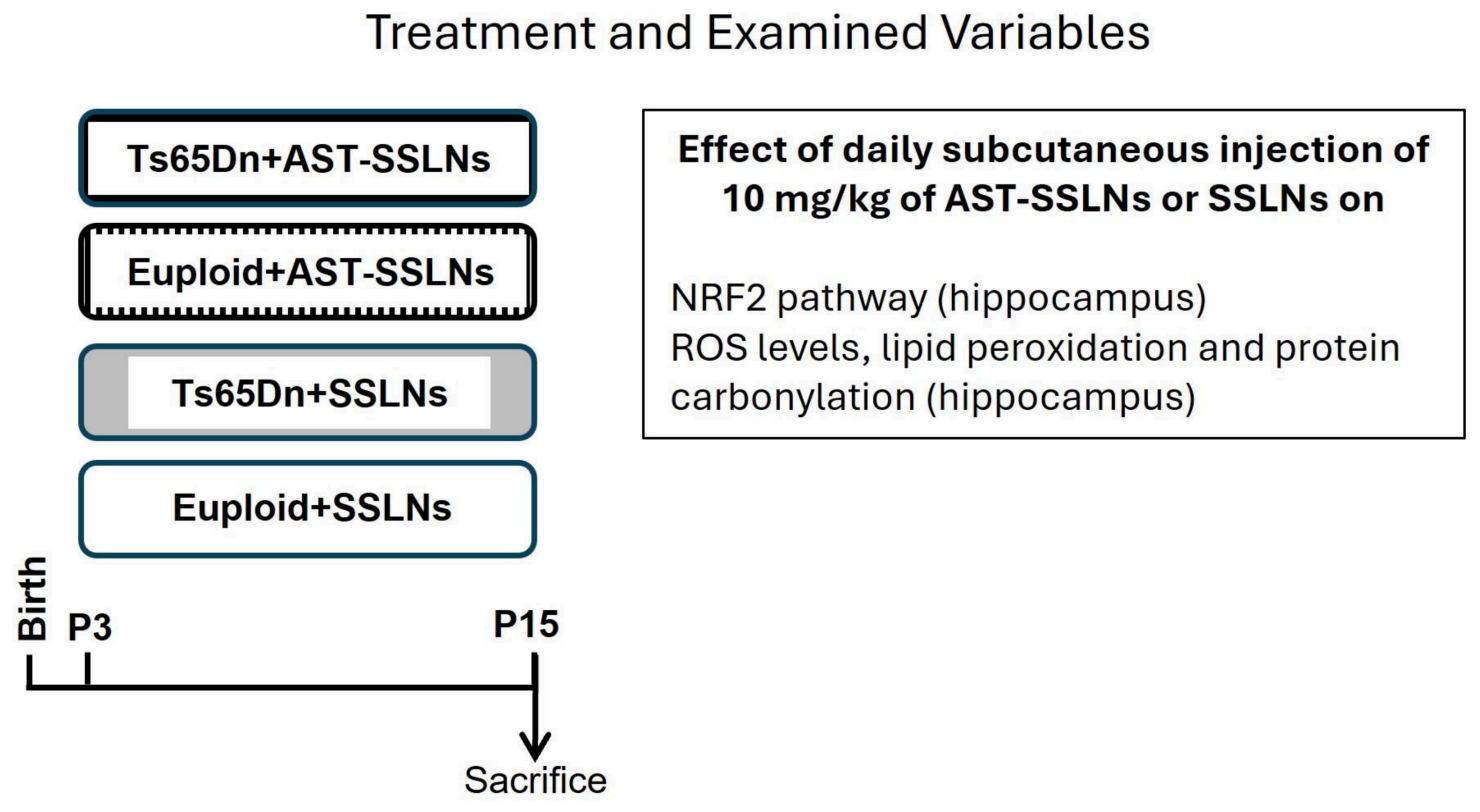
Experimental protocol: treatment and examined variables. Euploid and Ts65Dn received a daily injection of SSLNs or AST-SSLNs from P3 to P15. On P15, the brains were subjected to the analyses indicated in the panels on the right. Abbreviations: AST-SSLNs, Astaxanthin-loaded stealth solid lipid nanoparticles; NRF2, nuclear erythroid 2-related factor 2; P, postnatal; ROS, reactive oxygen species; SSLNs, stealth solid lipid nanoparticles.

The mice received a daily subcutaneous injection of AST-loaded SSLNs diluted in PBS corresponding to a dose of AST of 10 mg/kg or an equal volume of unloaded SSLNs from P3 to P15 (Figure 1). This is a critical period in mice for the development of the hippocampal dentate gyrus. The dose of 10 mg/kg was selected based on the literature data showing that this dose reduces oxidative stress and corrects cognitive deficits in different mouse models of central nervous system pathologies [26,27,40]. A total of 40 SSLN-treated mice (euploid mice *n* = 21; 8 females, 13 males; Ts65Dn mice *n* = 19; 8 females, 11 males) and 43 AST-SSLN-treated mice (euploid mice *n* = 23; 12 females, 11 males; Ts65Dn mice *n* = 20; 8 females, 12 males) were included in the analyses reported below.

The mice were deeply anesthetized with 2% isoflurane in oxygen and subsequently euthanized by an overdose of isoflurane. After confirmation of death, the brain was rapidly removed, weighed, and dissected along the midline. In a first group of mice, the right hemisphere was dissected into different brain regions to separate the hippocampi, which were kept at −80 °C and used for Western blot analysis, as described below. In a second group of mice, the right and left hemispheres were dissected to separate the hippocampi, which were kept at −80 °C and used for the detection of ROS levels and quantification of lipid peroxidation, respectively, as described below. In a third group of mice, the right hemispheres were dissected to separate the hippocampi, which were kept at −80 °C and used for the determination of protein carbonyl content, as described below.

### 2.4. Protein Sample Preparation

Hippocampi of untreated mice and treated mice from the first group were homogenized on ice in 100 ul of ice-cold RIPA buffer containing 50 mM Tris HCl (pH 8), 50 mM NaCl, 0.5% sodium deoxycholate, 1% Triton X-100, 0.5% sodium dodecyl sulfate, 1% protease inhibitor (#78437, Sigma-Aldrich, St. Louis, MO, USA), 1% phosphatase inhibitor (#P2850, Sigma-Aldrich, phenylmethanesulfonyl fluoride (1 mM, PMSF, #93482, Sigma-Aldrich), and milli-Q water to volume. After 30 min in ice, the samples were sonicated for 15 min and then centrifuged at 14,000× *g* rpm at 4 °C for 20 min to remove cellular debris. The supernatant was used to determine the total protein concentration using the Bradford method.

### 2.5. Western Blotting

An equivalent amount of protein (25 μg) was resolved on Bolt™ Bis-Tris Plus Mini Protein Gels, 4–12%, 1.0 mm, 17 wells (#NW04127BOX, Invitrogen, by Thermo Fisher Scientific, Waltham, MA, USA) and transferred to a Hybond ECL nitrocellulose membrane (#GE10600003, Amersham plc, Amersham, UK). The following primary antibodies were used: anti-BACH1 (1:1000, #orb4401, Biorbyt, Cambridge, UK); anti-NRF2 (1:1000, #GTX103322, GeneTex, Inc., North America, CA, USA); anti-p-NRF2 (Ser40, 1:1000, #PA5-67520, Invitrogen); anti-HO-1 (1:1000, #ADI-SPA-896-F, Enzo Biochem, Inc., Farmingdale, NY, USA); and anti-GAPDH (1:5000, #G9545, Sigma-Aldrich, Merck, Darmstadt, Germany). Detection was performed with an HRP-conjugated anti-rabbit (#111-035-003, Jackson Immunoresearch, West Grove, PA, USA) or anti-mouse (#115-035-003, Jackson Immunoresearch) secondary antibodies. Densitometric analysis of digitized images with ChemiDoc XRS+ was performed using Image Lab Software 3.0.1 Bio-Rad Laboratories, Hercules, CA, USA) and the intensity for each band was normalized to the intensity of the corresponding GAPDH band.

### 2.6. Detection of ROS Levels

The production of ROS was assessed using the oxidation-sensitive nonfluorescent dye 2′,7′-dichlorodihydrofluorescein diacetate (H2DCFDA). This molecule, in the presence of ROS such as H_2_O_2_, is oxidized to the fluorescent probe 2′,7′-dichlorofluorescein (DCF). Tissue samples of the hippocampal formation from animals in the second group were homogenized in 100 μL of 40 mM Tris HCl buffer (pH 7,4) on ice; then, the samples were sonicated for 5 min. Next, 45 μL of the hippocampal homogenates were incubated with H2DCFDA (10 μM final concentration; #D6883, Sigma Aldrich) at 37 °C for 40 min. Fluorescence was monitored on a GloMax Explorer Multimode Microplate reader (Promega Corporation, Madison, WI, USA) (λexcitation = 504 nm; λemission = 529 nm). Quantification of ROS was assessed from a standard curve of DCF (#D6665, Sigma Aldrich), ranging from 5 μM to 0.01 μM. Values were normalized by protein concentration, previously quantified using the Bradford method, and the results were expressed as μM of DCF produced per mg of protein.

### 2.7. Quantification of Lipid Peroxidation

Lipid peroxidation, a well-characterized mechanism of oxidative damage in cells, was measured by quantifying its end product, 4-hydroxynonenal (HNE). HNE is capable of binding to proteins and forming stable adducts, which were detected using the OxiSelect™ HNE Adduct Competitive ELISA Kit (#STA-338; Cell Biolabs, INC, San Diego, CA, USA). Briefly, the hippocampal formation from animals in the second group was homogenized in 100 μL of PBS 1X (pH 7.4) containing Protease Inhibitor Cocktail III (1:100, #539134, Merck Millipore, Darmstadt, Germany) on ice; then, the samples were sonicated for 5 min. The supernatant fraction was collected, and HNE levels were quantified using the HNE ELISA Kit (#STA-338; Cell Biolabs) following the manufacturer’s instructions. The absorbance of each sample was detected using a microplate reader (Optic Ivymen System, #2100-C, COMECTA, Barcelona, Spain). Values were normalized by protein concentration, previously quantified using the Bradford method, and the results were expressed as μg of HNE produced per mL.

### 2.8. Determination of Protein Carbonyl Content

Protein oxidative modification was evaluated in the hippocampus by quantifying carbonyl groups in proteins, as previously described by Levine et al., 1990 [41]. Briefly, hippocampi of animals from the third group were homogenized in 10 mM PBS (pH 7.4) containing Protease Inhibitor Cocktail III (1:250, #539134, Merck Millipore) on ice, followed by centrifugation at 14,000× *g* rpm for 15 min at 4 °C. After this, 100 µL from each sample were incubated with 10 mM 2,4-dinitrophenylhydrazine (DNPH) for 1 h at room temperature in the dark and precipitated with 20% TCA. Afterwards, the proteins were centrifuged (14,000× *g* rpm for 5 min) and washed thrice with ethanol:ethyl acetate (1:1 *v*/*v*). The proteins were then solubilized in 6 M guanidine (pH 6.0) and incubated for 60 min at 37 °C. The carbonyl content was measured at 360 nm using a microplate reader (Optic Ivymen System, #2100-C, COMECTA, Barcelona, Spain). Total protein concentration was determined, and carbonyl content was expressed as nmol carbonyl/mg protein.

### 2.9. Statistical Analysis

Results are expressed as mean ± standard error of the mean (SE). Statistical analyses were performed using IBM SPSS 22.0 software (IBM, Armonk, NY, USA). Before running statistical analyses, data distribution and homogeneity of variances for each variable were checked using the Shapiro–Wilk test and Levene’s test respectively. If the data were normally distributed and variance was homogeneous, statistical analysis was carried out using either the two-tailed Student’s *t*-test for comparisons between untreated euploid and Ts65Dn mice or a two-way ANOVA with genotype (euploid, Ts65Dn) and treatment (SSLNs, AST-SSLNs) as factors. Post hoc multiple comparisons were carried out using Fisher’s least significant difference (LSD) test. If the data were not normally distributed and variance was heterogeneous, transformations were made to achieve normality. If the transformed data did not achieve normality, statistical analysis was carried out using the Mann–Whitney U test or the Kruskal–Wallis test followed by the Mann–Whitney U test. Based on the “Box plot” tool available in SPSS Descriptive Statistics, in each analysis we excluded the extremes, i.e., values that are larger than 3 times the IQ range [x ≥ Q3 + 3 × (IQ); x ≤ Q1 − 3 × (IQ)]. The number of mice included in each analysis, as well as any exclusions, is provided in the figure legends. A *p*-value of ≤0.05 was considered statistically significant. Sex differences were assessed in all statistical analyses performed, and no significant sex effect was observed.

## 3. Results

### 3.1. Early Characterization of the NRF2 Pathway in Ts65Dn Mice

**Figure 2:**
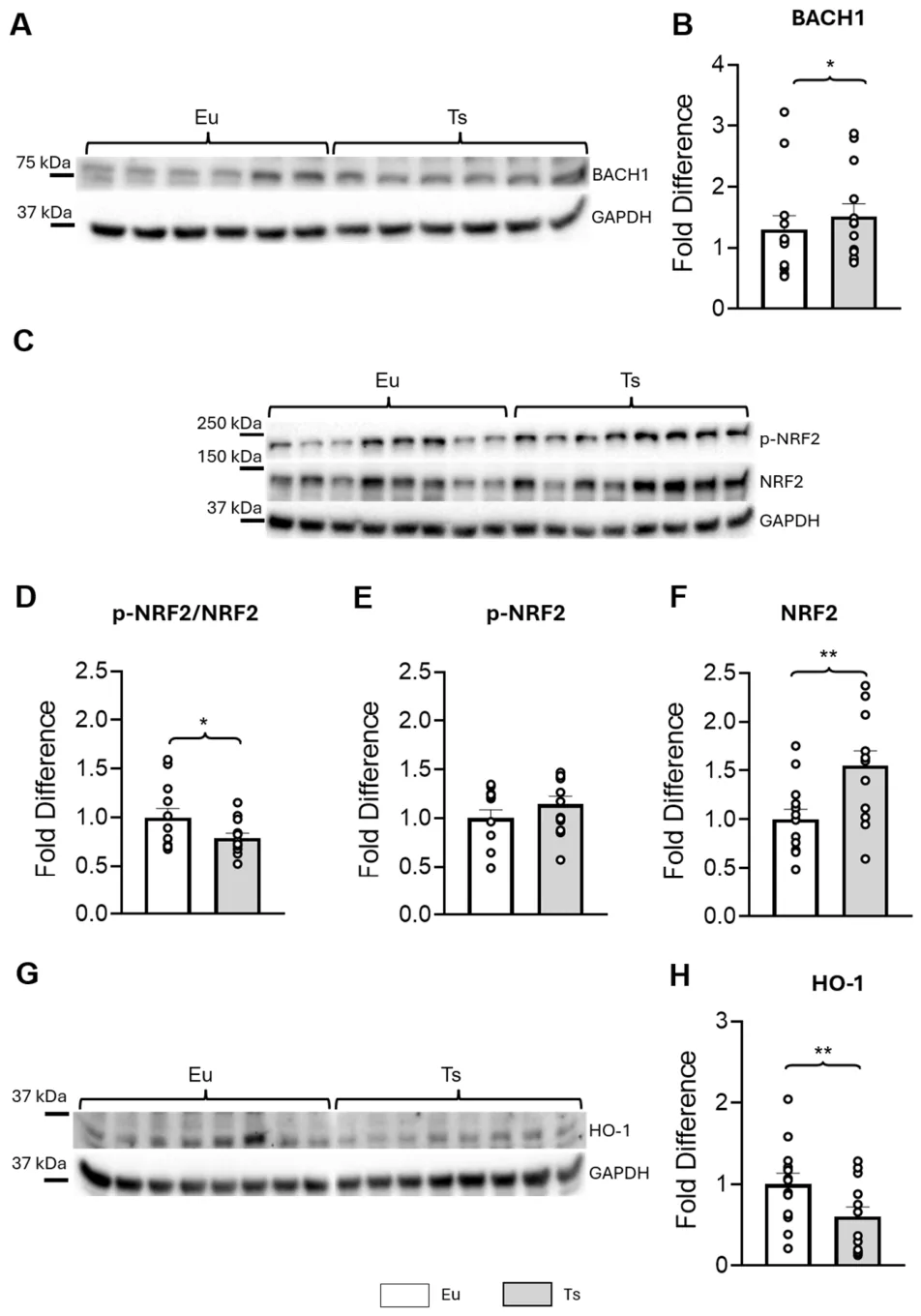
Characterization of the hippocampal NRF2 pathway in P15 euploid and Ts65Dn mice. Western blot analysis of BACH1, p-NRF2/NRF2, p-NRF2, NRF2, and HO-1 in the hippocampal formation of P15 euploid (*n* = 13; 5 females and 8 males) and Ts65Dn mice (*n* = 13; 4 females and 9 males). (**A**) Representative Western blot images showing immunoreactivity for BACH1 and the housekeeping gene GAPDH. (**B**) Levels of BACH1. (**C**) Representative Western blot images showing immunoreactivity for p-NRF2, NRF2 and the housekeeping gene GAPDH. (**D**–**F**) Levels of p-NRF2/NRF2 (**D**), p-NRF2 (**E**) and NRF2 (**F**). (**G**) Representative Western blot images showing immunoreactivity for HO-1 and the housekeeping gene GAPDH. (**H**) Levels of HO-1. Data in (**B**,**E**,**F**,**H**) were normalized to GAPDH; data in (**D**) were normalized to NRF2. Data (mean ± SE) are expressed as fold difference in comparison with untreated euploid mice. The scatterplots over each column (**B**,**D**,**E**,**F**,**H**) represent the values of individual cases for each group. * *p* ≤ 0.05; ** *p* ≤ 0.01. (Mann–Whitney test for data in B, Student’s *t*-test for data reported in (**D**–**F**,**H**)). Based on the statistical analysis, two euploid mice were excluded from BACH1 analysis. Abbreviations: BACH1, BTP Domain and CNC Homolog 1; Eu, euploid; GAPDH, glyceraldehyde-3-phosphate dehydrogenase; HO-1, heme oxygenase 1; NRF2, nuclear erythroid 2-related factor 2; p-NRF2, phosphorylated nuclear erythroid 2-related factor 2; Ts, Ts65Dn.

Since the NRF2 pathway has been shown to be impaired in trisomy 21 at adult life stages, resulting in a defective antioxidant response, we deemed it of interest to investigate the same pathway at an early developmental stage. For this purpose, we evaluated the levels of the different molecules involved in this signaling cascade in the hippocampal formation of untreated P15 euploid and Ts65Dn mice through WB analysis. A Mann–Whitney test on BACH1 levels showed a significant increase in this transcriptional factor in Ts65Dn mice compared to euploid mice (+52%; Figure 2A,B). A Student’s *t*-test on the ratio between the phosphorylated form of NRF2 (p-NRF2^Ser40^; an index of nuclear translocation [19]) and total NRF2 revealed that in untreated Ts65Dn mice, there is a reduction in this parameter compared to untreated euploid mice (−21%; Figure 2C,D). Regarding p-NRF2 total levels, a Student’s *t*-test revealed no difference between groups (Figure 2C,E). A Student’s *t*-test on total NRF2 levels showed a significant increase in NRF2 levels in Ts65Dn mice in comparison with euploid mice (+55%; Figure 2C,F). The NRF2 pathway modulates the expression of important antioxidant enzymes in response to cellular OS levels. One of the enzymes regulated by NRF2 that has been found to be impaired in trisomy 21 is HO-1 [19]. For this reason, hippocampal levels of HO-1 in euploid and Ts65Dn mice were investigated. A Student’s *t*-test showed a significant reduction in HO-1 levels in the untreated trisomic group compared to the euploid one (−41%; Figure 2G,H).

Taken together, these results show an early defective activation of the NRF2 pathway in Ts65Dn mice in comparison with the euploid group, ultimately leading to a reduced expression of HO-1, a crucial enzyme in the cellular response against OS.

### 3.2. Effect of Neonatal Treatment with AST-SSLNs on the NRF2 Pathway in the Hippocampal Formation of Ts65Dn and Euploid Mice

**Figure 3:**
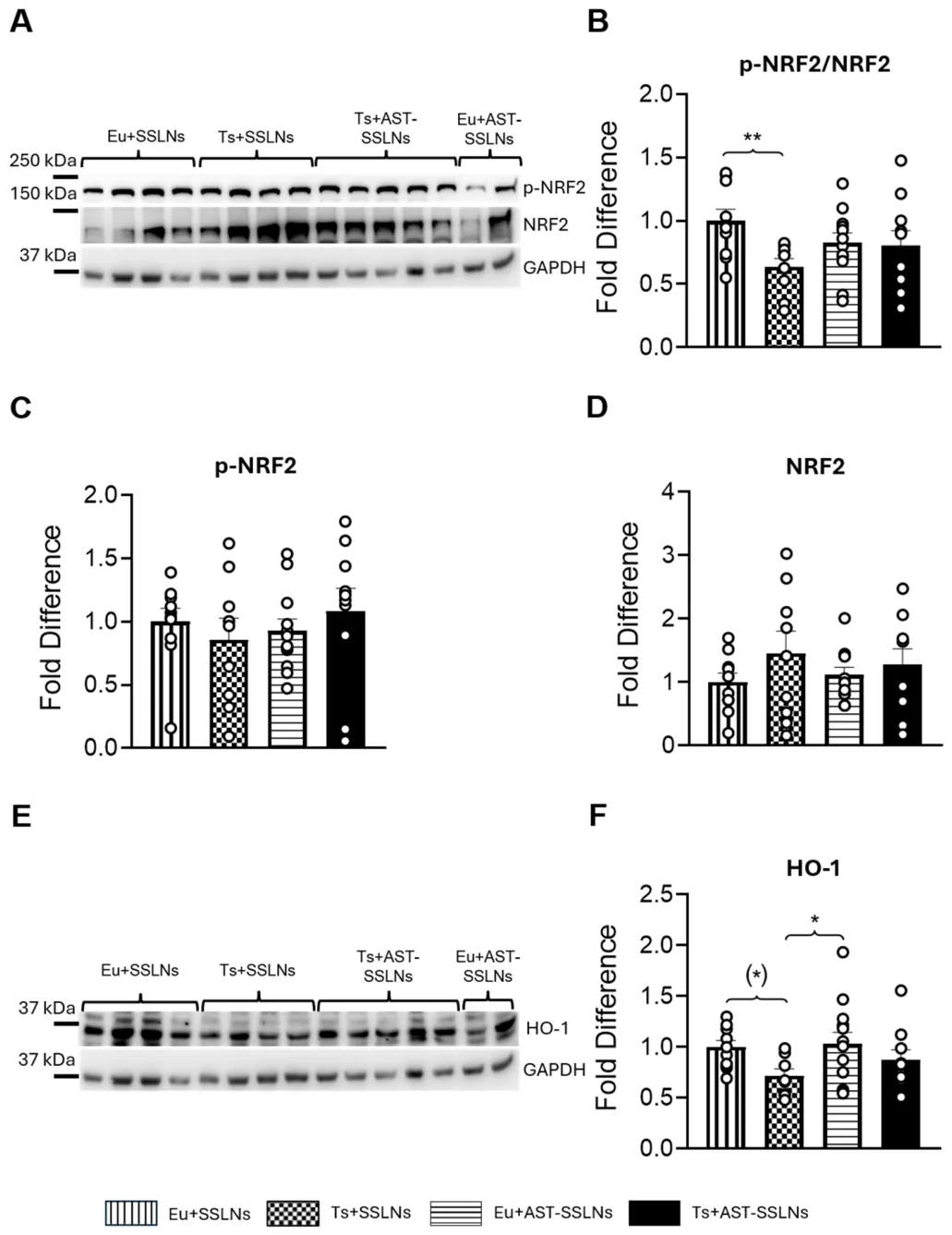
Effect of neonatal treatment with AST-SSLNs on the hippocampal NRF2 pathway in P15 euploid and Ts65Dn mice. Western blot analysis of p-NRF2/NRF2, p-NRF2, NRF2 and HO-1 in the hippocampal formation of P15 euploid (*n* = 10; 5 females and 5 males) and Ts65Dn mice (*n* = 9; 4 females and 5 males) treated with SSLNs and euploid (*n* = 13; 6 females and 7 males) and Ts65Dn mice treated with AST-SSLNs (*n* = 10; 4 females and 6 males). (**A**) Representative Western blot images showing immunoreactivity for p-NRF2, NRF2 and the housekeeping gene GAPDH. (**B**–**D**) Levels of p-NRF2/NRF2 (**B**), p-NRF2 (**C**) and NRF2 (**D**). (**E**) Representative Western blot images showing immunoreactivity for HO-1 and the housekeeping gene GAPDH. (**F**) Levels of HO-1. Data in (**C**,**D**,**F**) were normalized to GAPDH, while data in (**B**) were normalized to NRF2. Data (mean ± SE) are expressed as fold differences in comparison with SSLN-treated euploid mice. The scatterplots over each column in (**B**–**D**,**F**) represent the values of individual cases for each group. (*) *p* = 0.06; * *p* ≤ 0.05; ** *p* ≤ 0.01. (Fisher’s LSD test after two-way ANOVA for data reported in (**B**,**C**); Mann–Whitney test after Kruskal–Wallis test for data reported in (**D**,**F**)). Based on the statistical analysis, one SSLN-treated Ts65Dn mouse was excluded from HO-1 analysis, and one AST-SSLN-treated euploid mouse was excluded from the analysis of p-NRF2 and NRF2 levels. Abbreviations: AST, astaxanthin; AST-SSLNs, astaxanthin-loaded solid lipid nanoparticles; Eu, euploid; GAPDH, glyceraldehyde-3-phosphate dehydrogenase; HO-1, heme oxygenase 1; NRF2, nuclear erythroid 2-related factor 2; p-NRF2, phosphorylated nuclear erythroid 2-related factor 2; SSLNs, stealth solid lipid nanoparticles; Ts, Ts65Dn.

Given the early impairment of the NRF2 pathway in trisomy 21, we evaluated whether neonatal treatment with AST-SSLNs could ameliorate this defect. To this purpose, we examined the NRF2 pathway in the hippocampal homogenates of euploid and Ts65Dn mice that were daily treated with SSLNs or AST-SSLNs from P3 to P15. First, we established whether unloaded SSLNs could affect the NRF2 pathway. In euploid and Ts65Dn mice treated with SSLNs, we found no significant difference in p-NRF2/NRF2, p-NRF2, and total NRF2 in comparison with their untreated counterparts. Therefore, in the following statistical analyses, the levels of these proteins included euploid and Ts65Dn mice treated with SSLNs and euploid and Ts65Dn mice treated with AST-SSLNs. A two-way ANOVA on the ratio between the phosphorylated form of NRF2 (p-NRF2^Ser40^) and total NRF2 revealed a significant main effect of genotype [F(1,37) = 4.580, *p* = 0.039], but no main effect of either treatment or genotype × treatment. Post hoc Fisher’s LSD test revealed that in SSLN-treated Ts65Dn mice, the ratio between p-NRF2^Ser40^ and NRF2 was significantly lower compared to SSLN-treated euploid mice (−28%; Figure 3A,B), confirming the basal difference observed between untreated euploid and Ts65Dn mice (Figure 2C,D). An increase in this ratio was detected in AST-SSLN-treated Ts65Dn mice in comparison with SSLN-treated Ts65Dn mice (+26%; Figure 3A,B), although this increase was not statistically significant. After treatment, however, Ts65Dn mice had no longer a difference in this ratio in comparison with SSLN-treated euploid mice (Figure 3A,B), suggesting that treatment had normalized NRF2 pathway activation. A moderate decrease in the ratio between p-NRF2^Ser40^ and NRF2 was found in AST-SSLN-treated euploid mice compared to SSLNs euploid mice (−17%; Figure 3A,B); however, this difference was not statistically significant. A two-way ANOVA on total p-NRF2 levels showed no genotype × treatment interaction, no main effect of genotype and no main effect of treatment, revealing no statistically significant differences between groups (Figure 3A,C). The Kruskal–Wallis test on total NRF2 levels showed no significant difference between groups [χ^2(3)^ = 0.845, *p* = 0.839]. However, it is interesting to note that the NRF2 levels in SSLN-treated Ts65Dn mice were higher (+45%; Figure 3A,D) compared to SSLN-treated euploid mice, as previously demonstrated in untreated euploid vs. untreated Ts65Dn mice (Figure 2C,F). Since the activation of the NRF2 pathway induces the transcription of the antioxidant enzyme HO-1, we investigated whether treatment with AST could affect the hippocampal levels of this protein. A two-way ANOVA on the levels of HO-1 showed a significant main effect of genotype [F(1,37) = 5.118, *p* = 0.030], but no main effect of either treatment or genotype × treatment. Post hoc Fisher’s LSD test revealed that in SSLN-treated Ts65Dn mice, there was a marginally significant reduction in HO-1 levels compared to SSLN-treated euploid mice (−28%; Figure 3E,F), in agreement with data obtained in untreated euploid and Ts65Dn mice (Figure 2G,H). Treatment with AST caused an increase in HO-1 levels in Ts65Dn mice compared to SSLN-treated Ts65Dn mice (+22%), and these levels became statistically similar to those of SSLN-treated euploid mice (Figure 3E,F). Treatment with AST-SSLNs had no effect on HO-1 levels in the euploid group (Figure 3E,F).

Taken together, these findings show that neonatal treatment with AST-SSLNs can activate the impaired NRF2 pathway in Ts65Dn mice, with a consequent increase in the levels of the antioxidant enzyme HO-1.

### 3.3. Effect of Neonatal Treatment with AST-SSLNs on Oxidative Stress Levels in the Hippocampal Formation of Ts65Dn and Euploid Mice

**Figure 4:**
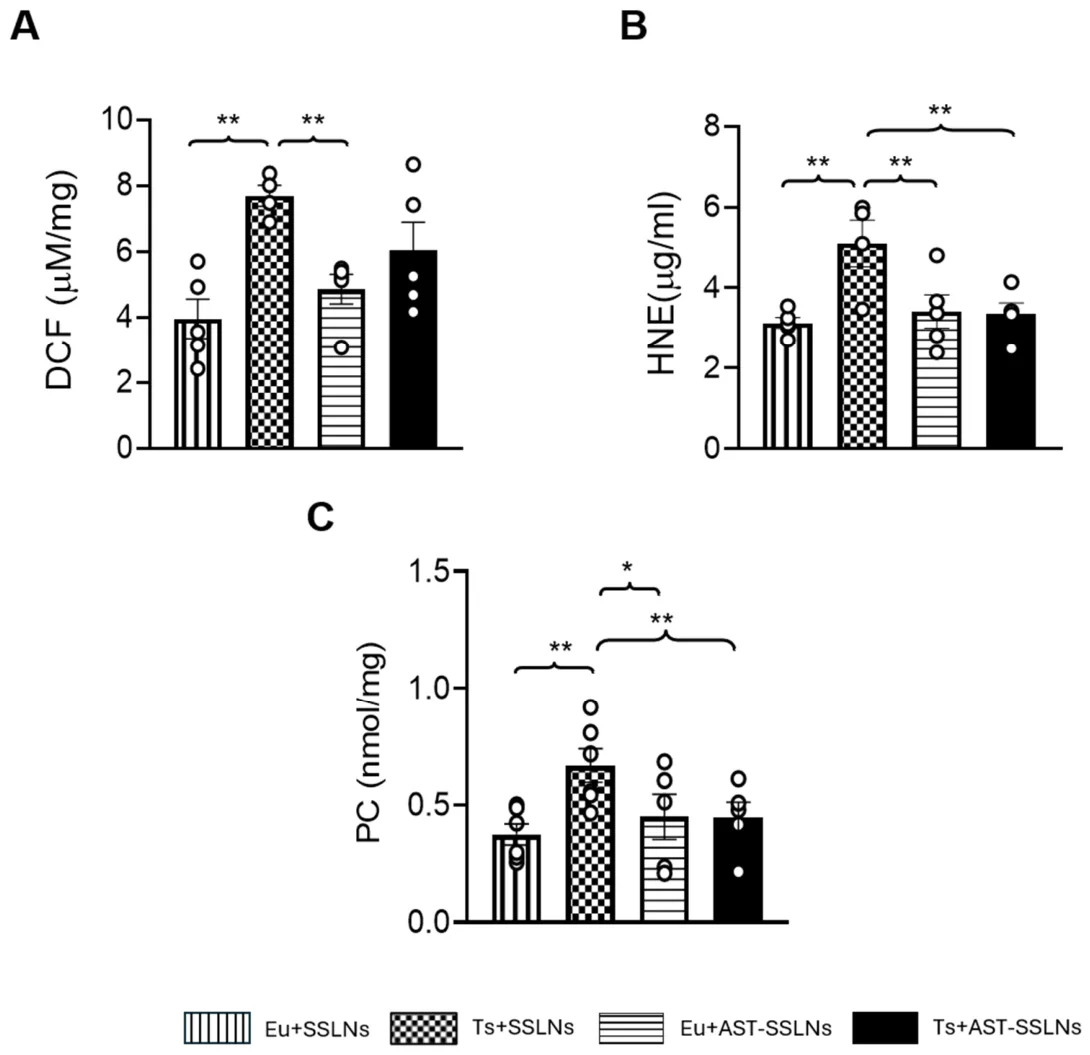
Effect of neonatal treatment with AST-SSLNs on hippocampal ROS levels, lipid peroxidation and protein carbonyl content in P15 euploid and Ts65Dn mice. Evaluation of DCF levels, HNE levels and protein carbonyl content in the hippocampal formation of P15 euploid and Ts65Dn mice treated with SSLNs, as well as euploid and Ts65Dn mice treated with AST-SSLNs. (**A**) ROS levels of euploid (*n* = 5; males) and Ts65Dn mice (*n* = 4; 1 female and 3 males) treated with SSLNs and euploid (*n* = 5; 3 females and 2 males) and Ts65Dn (*n* = 5; 2 females and 3 males) mice treated with AST-SSLNs. (**B**) HNE levels of euploid (*n* = 5; males) and Ts65Dn mice (*n* = 4; 1 female and 3 males) treated with SSLNs and euploid (*n* = 5; 3 females and 2 males) and Ts65Dn (*n* = 5; 2 females and 3 males) mice treated with AST-SSLNs. (**C**) Protein carbonyl content of euploid (*n* = 6; 3 females and 3 males) and Ts65Dn mice (*n* = 6; 3 females and 3 males) treated with SSLNs and euploid (*n* = 5; 3 females and 2 males) and Ts65Dn mice (*n* = 5; 2 females and 3 males) treated with AST-SSLNs. Values (mean ± SE) were normalized to protein concentration and expressed as μM of DCF produced per mg of protein (**A**), as μg of HNE produced per mL of sample (**B**), or nmol of PC per mg of protein (**C**). The scatterplots over each column represent the values of individual cases for each group. ** *p* ≤ 0.01, * *p* ≤ 0.05 (Mann–Whitney test after Kruskal–Wallis test for data reported in (**A**); Fisher’s LSD test after two-way ANOVA for data reported in (**B**,**C**)). Abbreviations: AST, astaxanthin; AST-SSLNs, astaxanthin-loaded solid lipid nanoparticles; DCF: 2′,7′-dichlorofluorescein; Eu, euploid; HNE, 4-hydroxynonenal; PC, protein carbonyl content; SSLNs, stealth solid lipid nanoparticles; Ts, Ts65Dn.

Since neonatal treatment with AST-SSLNs induced activation of the NRF2 pathway, we sought to establish whether this effect translated into a reduction of hippocampal ROS levels. To this purpose, we analyzed ROS levels in hippocampal homogenates using an H2DCFDA assay. The Kruskal–Wallis test on hippocampal ROS production revealed a difference between groups [χ^2^(3) = 8.553, *p* = 0.036]. The Mann–Whitney tests showed that ROS levels were significantly higher (+94%) in SSLN-treated Ts65Dn mice compared to SSLN-treated euploid mice (Figure 4A). AST-SSLN-treated Ts65Dn mice underwent a reduction in ROS production (−21%) in comparison with SSLN-treated Ts65Dn mice, and their levels became statistically similar to those of SSLN-treated euploid mice (Figure 4A). Treatment with AST had no significant effect on ROS levels in euploid mice (Figure 4A).

Increased oxygen radicals result in increased oxidative damage to fundamental biological molecules, such as lipids, which undergo an oxidative process called lipid peroxidation, culminating in the production of HNE, capable of forming stable protein adducts. Through the evaluation of these HNE protein adducts, we quantified the effects of AST-SSLNs on lipid oxidative damage in the hippocampal homogenates of euploid and Ts65Dn mice. A two-way ANOVA on HNE levels showed a genotype × treatment interaction [F(1,15) = 7.852, *p* = 0.013], a main effect of genotype [F(1,15) = 7.094, *p* = 0.018], and a marginally significant main effect of treatment [F(1,15) = 4.058, *p* = 0.062]. Post hoc Fisher’s LSD test revealed a significant increase in lipid peroxidation in SSLN-treated Ts65Dn mice in comparison with SSLN-treated euploid mice (+63%; Figure 4B). Treatment with AST-SSLNs significantly decreased HNE levels in Ts65Dn mice (−34%; Figure 4B), making them statistically similar to those of SSLN-treated euploid mice. Treatment with AST-SSLNs had no effect on HNE levels in the euploid group (Figure 4B).

Protein carbonylation (PC) is an irreversible post-translational modification of proteins induced by OS. This process, caused by an excess of free radicals, alters protein structure and function, acting as a key marker of cellular damage. Given the increased OS status in trisomy 21, we evaluated whether the PC index was altered in Ts65Dn mice compared with euploid mice and whether treatment with AST-SSLNs could modulate this parameter. A two-way ANOVA on PC levels showed a genotype × treatment interaction [F(1,18) = 4.462, *p* = 0.049], a main effect of genotype [F(1,18) = 4.294, *p* = 0.053], and no main effect of treatment. Post hoc Fisher’s LSD test revealed a significant increase in protein carbonylation in SSNL-treated Ts65Dn mice in comparison with their euploid counterparts (+79%; Figure 4C). AST-SSLNs significantly decreased PC levels in Ts65Dn mice (−33%; Figure 4C), making them statistically similar to those of SSLN-treated euploid mice. Treatment with AST-SSLNs had no effect on PC levels in the euploid group (Figure 4C).

Taken together, these results indicate that neonatal treatment with AST-SSLNs reduces excessive hippocampal ROS in Ts65Dn mice and that this effect is accompanied by a significant reduction in lipid peroxidation and protein damage.

### 3.4. Effect of Neonatal Treatment with AST-SSLNs on Body and Brain Weight of Ts65Dn and Euploid Mice

The Ts65Dn strain is characterized by a high mortality rate both during gestation and in the early postnatal period, before weaning [42]. Considering the fragility of this strain, we deemed it important to establish whether chronic treatment with AST-SSLNs may adversely affect the viability and gross growth parameters of P15 Ts65Dn mice. To this purpose, we evaluated the mortality rate and body and brain weight. We found that 7.5% of SSLN-treated mice and 9.4% of AST-SSLN-treated mice died in the P3–P15 period. The similarity in the mortality rate across groups suggests that AST treatment has no adverse effects on mice health.

**Figure 5:**
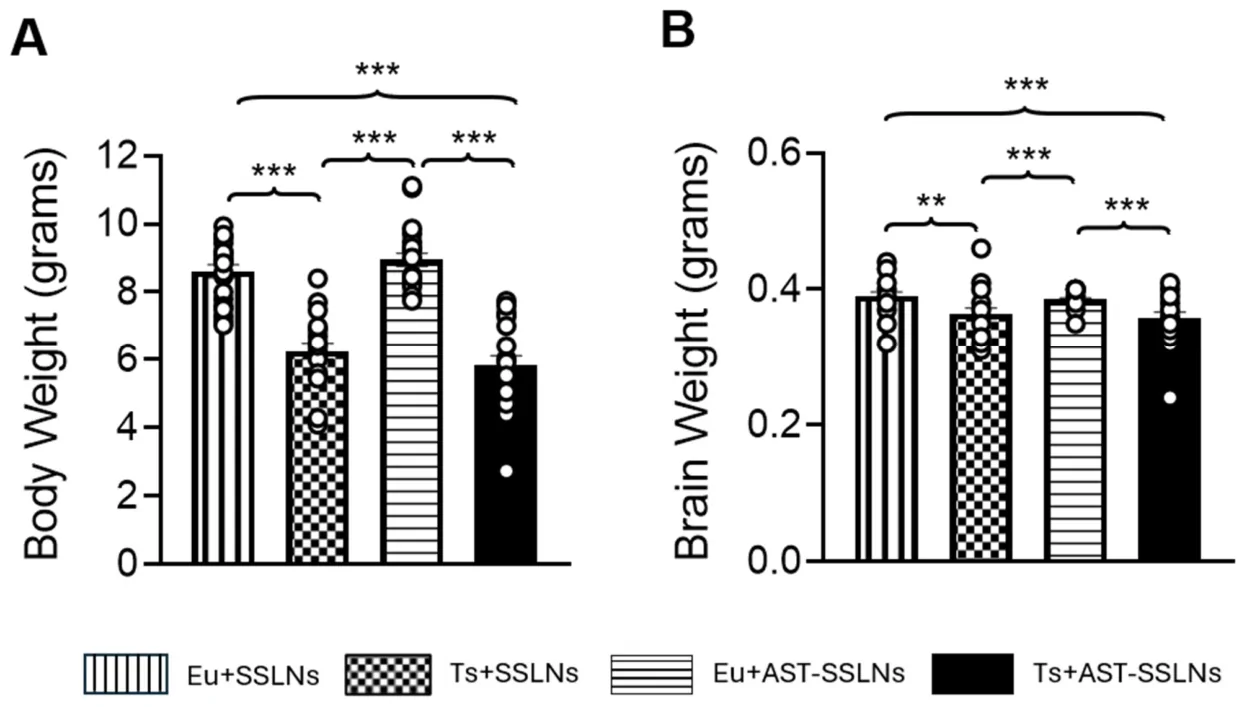
Effect of neonatal treatment with AST-SSLN on body and brain weight of P15 euploid and Ts65Dn mice. (**A**,**B**) Body (**A**) and brain (**B**) weight in grams of euploid (*n* = 21; 8 females and 13 males) and Ts65Dn (*n* = 19; 8 females and 11 males) mice treated with SSLNs and euploid (*n* = 23; 12 females and 11 males) and Ts65Dn (*n* = 20; 8 females and 12 males) mice treated with AST-SSLNs. Values represent mean ± SE. The scatterplots over each column represent the values of individual cases for each group. *** *p* ≤ 0.001, ** *p* ≤ 0.01 (Fisher’s LSD test after two-way ANOVA for data reported in (**A**); Mann–Whitney test after Kruskal–Wallis test for data reported in (**B**)). Abbreviations: AST, astaxanthin; AST-SSLNs, astaxanthin-loaded solid lipid nanoparticles; Eu, euploid; SSLNs, stealth solid lipid nanoparticles; Ts, Ts65Dn.

A two-way ANOVA on body weight showed no genotype × treatment interaction, no main effect of treatment, but a main effect of genotype [F(1,79) = 146.882, *p* < 0.001]. Post hoc Fisher’s LSD test showed a significant reduction in body weight in SSLN-treated Ts65Dn mice compared to SSLN-treated euploid mice (−25%; Figure 5A). The same significant difference in body weight was retained in euploid and Ts65Dn mice treated with AST-SSLNs, with no further body weight loss in Ts65Dn mice (Figure 5A). A Kruskal–Wallis test on brain weight revealed a significant difference between groups [χ^2^(3) = 19.672, *p* = 0.000]. The Mann–Whitney test showed that SSLN-treated Ts65Dn mice had a reduced brain weight (−6%) in comparison with SSLN-treated euploid mice. This difference was maintained after AST-SSLN treatment in euploid and Ts65Dn mice (Figure 5B).

These data show that neonatal treatment with AST-SSLNs has no adverse effects on the viability, body weight or brain weight of Ts65Dn mice.

## 4. Discussion

### 4.1. Early Alterations of the NRF2 Pathway in the Hippocampus of Ts65Dn Mice

The NRF2 pathway is involved in the control of cellular redox homeostasis by critically regulating the cellular antioxidant response. Indeed, NRF2 is a transcriptional regulator involved in the expression of different genes encoding antioxidant enzymes, such as HO-1, NQO1, and several glutathione-related enzymes [32]. In the nucleus, the transcription repressor BACH1 competes with the KEAP1-NRF2-ARE signaling complex for the expression of these genes involved in cell stress response. Thus, the induction of gene expression depends on the critical balance between BACH1 and NRF2. Previous evidence showed that the NRF2 pathway is impaired in the brains of juvenile Ts2Cje mice [19] and in adults with DS [20].

Our study in the hippocampus of Ts65Dn mice provides novel evidence that the BACH1/NRF2 axis is already compromised during the neonatal period. We found that BACH1 levels were increased in neonatal Ts65Dn mice in comparison with euploid mice (Figure 2B). Regarding the NRF2 pathway, we found that total NRF2 levels were increased in trisomic mice, likely as a compensatory response to chronic oxidative stress (Figure 2F). This data is in agreement with data obtained in vitro in trisomy 21 human fibroblasts and mouse embryonic fibroblasts derived from Dp16 mice in response to increased OS [43]. We additionally found that the increase in NRF2 levels did not translate into an enhancement of NRF2 phosphorylation at Ser40, an index of NRF2 nuclear translocation and activation of the antioxidant response. This resulted in a reduced ratio between p-NRF2 and NRF2 in trisomic mice compared to euploid mice (Figure 2D). It is conceivable that NRF2 nuclear translocation is precluded by the higher BACH1 levels. Increased BACH1 levels, together with decreased NRF2 nuclear translocation, should result in a decreased transcription of antioxidant enzymes controlled by NRF2. Indeed, the analysis of the levels of one of these enzymes, HO-1, showed a reduction in Ts65Dn mice in comparison with euploid mice (Figure 2H).

Taken together, these preclinical data suggest that the BACH1/NRF2 imbalance is already present during the critical window of hippocampal development, impairing the brain’s endogenous capacity to counteract a compromised redox environment.

### 4.2. Treatment with AST-SSLNs Positively Modulates the Altered NRF2 Pathway and Reduces Oxidative Stress in the Hippocampus of Ts65Dn Mice

The current study shows that treatment with AST-SSLNs during the neonatal period is able to increase p-NRF2 levels in the hippocampus of Ts65Dn mice, resulting in a higher ratio between p-NRF2 and total NRF2 levels, with no significant differences compared with SSLN-treated euploid mice (Figure 3B). Activation of this pathway in trisomic mice treated with AST-SSLNs led to an increase in the antioxidant enzyme HO-1 to levels statistically similar to those of SSLN-treated euploid mice (Figure 3F). The action of AST on the NRF2 pathway has been extensively demonstrated in both in vivo and in vitro studies, showing that it decreases KEAP1 expression [44], preventing the sequestration and degradation of newly synthesized NRF2, and actively promotes the phosphorylation and nuclear import of NRF2, effectively stimulating the transcriptional activity of antioxidant enzymes, even in severely compromised redox environments [31,32]. Given the positive effect of AST-SSLN treatment on the impaired NRF2 pathway, we next evaluated its efficacy on the oxidative profile in the hippocampus of P15 mice. We found that treatment significantly reduced the excessive accumulation of ROS (Figure 4A). This effect was accompanied by a complete normalization of the increased lipid peroxidation and protein carbonylation, two classical indicators of ROS imbalance and oxidative injury (Figure 4B,C). Previous evidence showed higher ROS content, increased lipid peroxidation and higher levels of oxidized proteins in the hippocampal homogenates of adult Ts65Dn mice compared to euploid mice [45]. To our knowledge, this is the first evidence showing that ROS levels, lipid peroxidation and protein carbonyl content are already increased in the hippocampus of Ts65Dn mice during the early postnatal period and that treatment with AST-SSLNs is able to counteract these alterations. The beneficial effects of AST on OS may be underpinned not only by the positive modulation of the NRF2 pathway but also by its ability to directly scavenge ROS through its polyene chain and chemical quenching mechanisms [46].

The neonatal period represents a critical window for hippocampal development, particularly for neurogenesis and neuronal maturation in the dentate gyrus. AST-mediated attenuation of early-stage OS in trisomy 21 may be relevant for counteracting alterations in these neurodevelopmental processes that are highly sensitive to elevated OS. Notably, AST has been shown to improve hippocampal neurogenesis [47], long-term potentiation, and several hippocampal-dependent cognitive tasks [48] in wild-type mice. Moreover, AST was reported to partly recover dendritic spine loss in hippocampal neurons of a rat model of Alzheimer’s disease [49]. All this evidence suggests that the observed beneficial effects of AST-SSLNs on OS may be accompanied by restoration of hippocampal neurogenesis, dendritic maturation, and hippocampus-dependent memory in the Ts65Dn model of trisomy 21.

### 4.3. Treatment with AST-SSLNs Has No Adverse Effects on Viability and Growth of Ts65Dn Mice

In this study, we found that a 13-day-long neonatal treatment with AST-SSLNs, corresponding to a dose of AST of 10 mg/kg, did not affect mice viability and did not cause body or brain weight reduction in Ts65Dn mice (Figure 5), suggesting a safe profile for this innovative nanoformulation in this mouse strain. It must be noted that systemic administration of AST-SSLNs in a mouse model has been tested here for the first time. No toxic effects of these nanoparticles have been previously found in vitro in a stem cell line and in a cancer cell line [33].

Regarding the safety profile of AST, chronic dietary administration of this compound in mice has been reported to be well tolerated. In particular, a 30-day repeated-dose administration study showed that mice receiving AST at low, medium, and high doses (respectively 4.2, 8.35, and 16.70 mg/kg body weight/day) exhibited no adverse histological alterations in major organs, including the liver, kidney, spleen, and heart [50]. In humans, a safety report based on evidence from over 80 clinical studies has demonstrated the absence of severe adverse events and no indicators of liver toxicity using short-term daily doses (up to 100 mg) and long-term daily doses (up to 20–40 mg/day) of natural AST-containing supplements [51].

Additional studies are needed to rule out subtle side effects on specific organs and functions of the nanoformulation tested in this study.

### 4.4. Study Limitations

Some limitations of the present study should be acknowledged. The sample sizes available for some biochemical analyses, particularly those assessing oxidative stress markers reported in Figure 4, were relatively small. This limitation mainly reflects the relatively low number of Ts65Dn pups available across litters and the need to minimize the number of animals, in accordance with the principle of Reduction. A larger sample size would allow us to draw more robust conclusions regarding the effects of AST-SSLNs on these parameters and would also enable an assessment of potential sex-related differences in treatment efficacy. An additional limitation relates to the developmental stage examined. This study was specifically designed to investigate molecular and biochemical alterations during the early postnatal period, with analyses performed at P15. Since at this developmental stage, mice cannot be behaviorally tested with conventional hippocampus-dependent paradigms commonly used to assess learning and memory, such as the Morris water maze and passive avoidance test, the functional relevance of the observed biochemical improvements could not be examined here. Future studies specifically designed to assess whether the beneficial effects of AST-SSLN treatment, restricted to the P3–P15 developmental window, persist after treatment discontinuation, or whether prolonged treatment is required to maintain these effects at later life stages will be important. Such studies should also include the assessment of hippocampus-dependent behavioral outcomes, together with hippocampal development in terms of neurogenesis and dendritic maturation. These investigations will help determine whether the biochemical effects of AST-SSLN treatment observed here translate into persistent structural and functional improvements.

## 5. Conclusions

This study provides novel preclinical evidence that the antioxidant NRF2 pathway is impaired in trisomy 21 from the neonatal period, a critical window for the development of the hippocampal dentate gyrus, suggesting that this early postnatal period may represent a valuable therapeutic window to counteract oxidative stress.

We additionally demonstrated that neonatal systemic administration of AST-SSLNs activates the impaired NRF2 pathway, and this effect translates into a full normalization of oxidative damage markers, reducing excessive ROS accumulation and reversing both lipid peroxidation and protein damage in the trisomic hippocampal tissue.

These findings suggest that the administration of this nanoformulation could represent a promising and innovative tool for the treatment of OS in DS.

## Data Availability

The raw data supporting this study are available from the corresponding author upon reasonable request, as they form part of an ongoing research project and cannot be publicly shared at this time.

## Abbreviations

The following abbreviations are used in this manuscript:

AST	Astaxanthin
APP	Amyloid precursor protein
AREs	Antioxidant response elements
AST-SSLNs	Astaxanthin-loaded stealth solid lipid nanoparticles
BACH1	BTB and CNC Homology 1
DCF	2′,7′-dichlorofluorescein
DNPH	2,4-dinitrophenylhydrazine
DS	Down syndrome
EU	Euploid
H2DCFDA	2′,7′-dichlorodihydrofluorescein diacetate
HNE	4-hydroxynonenal
HO-1	Heme oxygenase-1
HSA21	Human chromosome 21
KEAP1	Kelch-like ECH-associated protein 1
NADPH	Nicotinamide adenine dinucleotide phosphate
NQO1	Quinone oxidoreductase 1
NRF2	Nuclear factor erythroid 2-related factor 2
OS	Oxidative stress
P	Postnatal day
PC	Protein carbonyl content
PDI	Polydispersity index
ROS	Reactive oxygen species
S100 β	S100 beta
SOD1	Superoxide dismutase 1
SSLNs	Stealth solid lipid nanoparticles
TS	Ts65Dn mouse
Z-Ave	Average particle size
ZP	Zeta potential
